# Ferd. C. Valentine

**Published:** 1893-02

**Authors:** 


FERD. C. VALENTINE.

---

BUFFALO, January 4, 1893.

*Dr. F. C. Valentine :*

As I am not a subscriber to the BUFFALO MEDICAL AND SURGICAL JOURNAL, I cannot say whether all you charge me with is true. The paper was read at a society meeting, when it was announced that it was not origintal *in toto*, and that as much of it as was my own was already in print, save a case report, and that it was read by request of others, not on my own volition.

The portion you so strenuously object to *was* a cutting from your journal, I grant. At the conclusion of the evening, the assistant editor of the above journal asked me to let him take it to summarize it. I was quite surprised to find it practically reprinted, and though I have seen the proof—hurriedly—have not seen it in page form. If I remember anything about it in this connection, it is that credit is given to the *sources* whence came what was copied, though I don't recall that your paper is mentioned. I owe it to myself to say that this was not an intentional slight, but that I never supposed what I *read* would appear in print.

But, in saying this, I have done my duty, and am in no wise responsible for what a journal may do with matter not originally intended for publication. Your earnestness amuses me, and I like it, but your endeavor to make of me purely an advertising medium is not to my taste, nor do I propose to be responsible for statements as to the money value of what your journal does or purports to represent. You will never find me slow to repair a wrong or to rectify an unintentional error, but you will find certain traits in my character less pleasing to you should you provoke their exhibition. A quotation from your journal should be regarded as a compliment to it, not as an insult. Did you anticipate that I could have stated its circulation, its advantages, etc., providing I had alluded to it at all, without previous correspondence with yourself ?

But, seriously, I see how you feel, and am sorry that the accident should have so annoyed you. When you have done more work of the kind you are doing so well, you will hear and see many slips of tongue and pen, *in others*, which you deprecate. Some such slip you will then be convinced you made in writing such a letter to me. Had you suggested an *amende honorable*, I should have been pleased to comply. What you propose, however, is far more dishonorable to yourself than my fault of omission, of which you justly complain.

Should the editors of the BUFFALO JOURNAL permit it, and you desire it, I have no objection to the appearance in its pages of both our letters, yours and mine.

Yours truly, ROSWELL PARK.

---

JANUARY 7, 1893.

*Dr. Roswell Park, 510 Delaware avenue, Buffalo, N. Y.:*

DEAR DOCTOR—I accept your proposition to have your and my letters appear in the BUFFALO MEDICAL AND SURGICAL JOURNAL, and shall look to the February issue of that publication for settlement of the question.

Yours truly, FERD. C. VALENTINE.

---

THE lower berth of a Pullman sleeper contains forty-five to fifty cubic feet of air, hemmed in with walls on all sides, and little opportunity for its renewal (*South. Clinic*). Pure air contains one-fifth oxygen. There would then be in the berth ten cubic feet of oxygen. A healthy man inhales sixteen cubic feet of oxygen every twenty-four hours, and exhales fourteen cubic feet of carbon dioxide. If he occupies such a berth from 9 P. M. to 7 A. M. he would abstract from it six cubic feet of oxygen and inject into it six cubic feet of carbon dioxide, and various effete products of the body. Instead of seven parts of carbon dioxide per 10,000, the full amount allowed from exhalation to a healthy atmosphere, before he left his sleeping apartment in the morning, it would contain over 1,000 parts per 10,000. Alas! for the luxury of a sleeping car.—*Medical Review* (*St. Louis*).

---

PAPER money may be a dangerous medium for the transmission of pathogenic bacteria. Two notes examined by experts in Havana yielded more than 19,000 germs of various kinds. Cultures of these injected into the peritoneal cavity of guinea-pigs caused death, in almost every instance, in twenty-four hours.—*Med. Bul.* (*Chicago.*)

